# Development of a Method to Extract Opium Poppy (*Papaver somniferum L*.) DNA from Heroin

**DOI:** 10.1038/s41598-018-20996-9

**Published:** 2018-02-07

**Authors:** Michael A. Marciano, Sini X. Panicker, Garrett D. Liddil, Danielle Lindgren, Kevin S. Sweder

**Affiliations:** 1 Forensic & National Security Sciences Institute, Syracuse University, Syracuse, New York 13244 USA; 2 U.S. Drug Enforcement Administration, Special Testing and Research Laboratory, Dulles, VA 20166 USA

## Abstract

This study is the first to report the successful development of a method to extract opium poppy (*Papaver somniferum L*.) DNA from heroin samples. Determining of the source of an unknown heroin sample (forensic geosourcing) is vital to informing domestic and foreign policy related to counter-narcoterrorism. Current profiling methods focus on identifying process-related chemical impurities found in heroin samples. Changes to the geographically distinct processing methods may lead to difficulties in classifying and attributing heroin samples to a region/country. This study focuses on methods to optimize the DNA extraction and amplification of samples with low levels of degraded DNA and inhibiting compounds such as heroin. We compared modified commercial-off-the-shelf extraction methods such as the Qiagen Plant, Stool and the Promega Maxwell-16 RNA-LEV tissue kits for the ability to extract opium poppy DNA from latex, raw and cooked opium, white and brown powder heroin and black tar heroin. Opium poppy DNA was successfully detected in all poppy-derived samples, including heroin. The modified Qiagen stool method with post-extraction purification and a two-stage, dual DNA polymerase amplification procedure resulted in the highest DNA yield and minimized inhibition. This paper describes the initial phase in establishing a DNA-based signature method to characterize heroin.

## Introduction

The heroin epidemic has become a widespread domestic issue for the American people; it has touched the lives of families who have lost loved ones to heroin overdoses and is ubiquitous in the mainstream media. The heroin epidemic shows no signs of slowing. The number of individuals using heroin approached one million by the end of 2014, an increase from previous years^1^. U.S. officials have prioritized this issue through legislation and the development and continued support with governmental agencies. Such agencies include law enforcement, diversion control, and interdiction programs such as the High Intensity Drug Trafficking Areas (HIDTA) program, Organized Drug Enforcement Task Force (OCDETF), the Heroin Signature Program and the Heroin Domestic Monitoring Program. Investigative and intelligence information regularly compiled by such entities enable the U.S. government to better understand drug traffic patterns to combat the heroin epidemic. In addition, the opium poppy cultivation and heroin production in various source countries are also being investigated.

Opium poppy (*Papaver somniferum L*.), a medicinal plant known to the human race since the ancient civilizations, continues to be cultivated around the globe for the production of pharmaceutical opiates and heroin. While the poppy straw harvested from cultivation is generally employed by pharmaceutical entities, the liquid or dried sap (known as opium latex or gum that is obtained by lancing the outer surface of poppy pods) is the starting material for the clandestine production of heroin. The research into opium alkaloid chemistry began with the isolation of morphine in the early 1800s. Several publications cite the presence of 80 or more alkaloids present in opium. The alkaloid composition of opium can be varied because of agronomical, climatic, or cultivar differences. The U.S. Drug Enforcement Administration’s Special Testing and Research Laboratory utilizes the differences in opium alkaloid profiles of various varieties of opium poppy (unpublished research) as the foundation for the Heroin Signature Program (HSP). The HSP involves four diverse signature methodologies that are independently employed^2–4^ (unpublished research at the laboratory on Isotope Ratio Mass Spectrometry). The results of the analysis will allow the sample to be classified by geographic region of origin: Mexico (MEX), South America (SA), Southwest Asia (SWA), or Southeast Asia (SEA).

Despite the significance of poppy varieties and their unique alkaloid profiles in the HSP, there have not been efforts to develop a signature method using opium poppy DNA. The chemical processing steps involved in producing heroin from opium require extreme alkaline and acidic pHs and high temperatures As a result, heroin samples are expected to contain exceedingly limited amounts of damaged and degraded poppy DNA. The lack of development efforts were likely due to the low likelihood of obtaining a suitable DNA signature. However, the genetic profiling of opium poppy remained a topic of great interest to stakeholders such as the Drug Enforcement Administration Special Testing and Research Laboratory^5–11^. Advancements in DNA amplification and sequencing technologies in the last decade prompted an innovative scientific investigation into utilizing the poppy DNA fragments detected in heroin as a classification tool.

The genetic characterization of *Papaver Somniferum L*. (opium poppy) has largely focused on the understanding and exploitation of the metabolic pathways that influence alkaloid production^12–17^. The genesis of this applied work relied on foundational genomic research that began characterizing the opium poppy. This has been central to the ability to exploit the opium poppy DNA for use in determining population-based genetic identity. The opium poppy was identified by Kadereit (1986) as an ancient triploid that has since evolved into a diploid genome with 11 chromosomes (2n = 22)^18–20^. The diploid nature of the poppy genome imparts a much simpler workflow for genetic identification, specifically because it mirrors the approaches used for human genetic identification using single copy DNA sequences. Polymorphic markers such as ISSRs and RAPDs were identified and used to demonstrate the lack of genetic diversity of legally cultivated opium poppy in India^20^. Masárová *et al*. further characterized variation in the opium poppy genome by mining all opium poppy sequence available through GenBank^21,22^. The purpose of this study was to identify and characterize microsatellite nucleotide sequences based on repeat composition and abundance in the genome. It permitted further research that focused on various aspects of genetic identification of the opium poppy. Although these microsatellites are of potential use for geosourcing opium poppy, we needed to mine the genome to ensure the microsatellites and single nucleotide polymorphisms had significant levels of variation among the relevant populations to permit geosourcing.

The development of DNA markers (microsatellites) to geosource heroin began by conducting pilot studies into opium poppy seeds/plants and opium samples (unpublished research). This manuscript presents the first results attempting to employ opium poppy genetics for the classification of heroin. While the identification of variety-specific and geography-specific DNA markers continues as part of an elaborate scheme of poppy genome research, the optimization of a DNA extraction method from crudely manufactured black tar and highly refined powder heroin samples, and its subsequent amplification are presented here.

## Results

### DNA Isolation: Heroin

Isolation of *Papaver somniferum L*. DNA from heroin presents a new and increasingly complex challenge compared to that encountered in the extraction process for opium. Heroin samples are inherently diverse. They vary in purity, cutting agents, level of processing and storage conditions. These factors have a significant impact on the resulting quality and quantity of opium poppy DNA obtained from the sample. Extraction methods that were evaluated include Commercial-off-the-shelf (COTS) methods: (1) an automated Promega Maxwell 16 Tissue RNA LEV silica/magnetic bead-based method^23^ and (2) the Qiagen Plant DNeasy silica column-based DNA extraction method and (3) a modified version of the Qiagen Stool kit DNA extraction method (Table 1). The study design considers several factors that may affect DNA recovery from heroin samples: (1) purity of heroin, (2) quantity of sample (grams), (3) extraction method, and (4) type of heroin sample (white or brown powder or black tar). Heroin samples with sufficiently high quantities (total mass) were selected to enable consistent comparisons across extraction methods (Table 2).

**Table 1:** DNA extraction methods used to evaluate success in extracting DNA from Heroin. *Note: The RNA low elution volume method has been adapted for use in DNA extractions.

Method	Lysis Method	Additional treatments	DNA purification medium	Execution
Qiagen Stool	ProK, heat, chaotropic salt	InhibitEX buffer	Silica column	Manual
Qiagen Plant	ProK, heat, chaotropic salt	QIAShredder column (Physical disruption)	Silica column	Manual
Maxwell Tissue RNA LEV*	ProK, heat, chaotropic salt	—	Silica-Magnetic Bead (MagneSil®PMPs)	Maxwell 16

*Note: The RNA low elution volume method has been adapted for use in DNA extractions.

**Table 2:** Heroin Samples selected for the initial evaluations of DNA extraction methods.

Sample	Description	% Heroin (HCl or Base)
WP-1	White Powder	86.4 (HCl)
BPc-1	Brown Powder (Coarse)	55.7 (HCl)
BPf-1	Brown Powder (Fine)	12.1 (Base)

**Figure 1:**
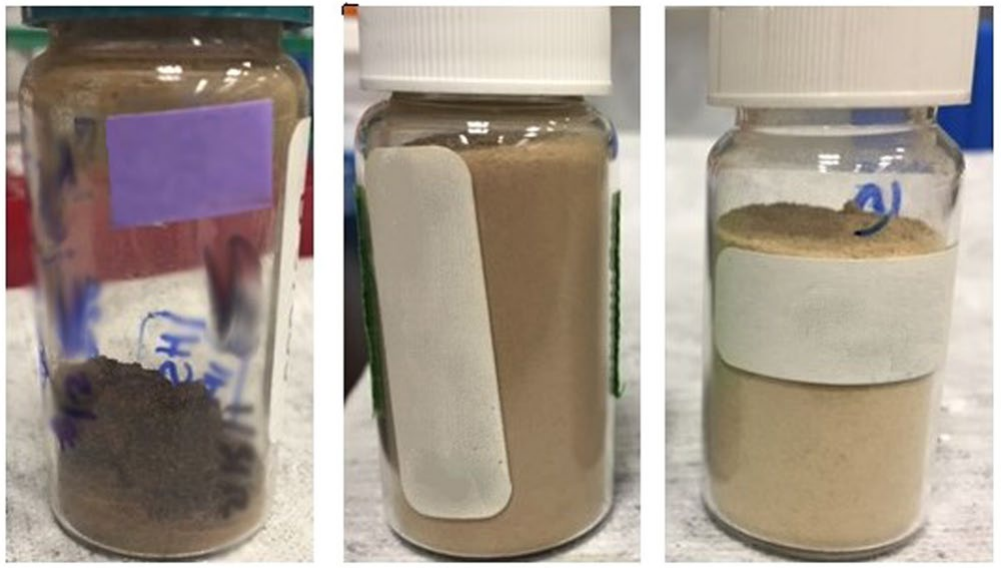
Heroin samples BT-2-black tar, BPf-1-brown powder (fine) and BPc-1-brown powder (coarse). Note, no picture of WP-1 is available.

The input mass of heroin samples were evaluated to determine the minimum mass required for significant DNA yield. The samples consisted of a white powder heroin-HCl (86.4% pure), brown powder heroin-HCl (55.7% pure), and brown powder heroin-base (12.1% pure). Each extraction method was evaluated using approximately 3.0 g of each sample, split between 2 tubes of 2.0 g and 1.0 g of heroin (Table 3 and Fig. 1). The white powder heroin sample (WP-1) was the only sample in sufficient quantity to permit an evaluation of the initial lysis step for the Maxwell Plant RNA LEV extraction method. The remaining samples did not have the sufficient quantity of material to perform this evaluation. (Note, individual heroin samples were limiting and many times did not have the sample mass to permit many replicates for comparison. This limited our ability to compare the extraction methods and unequivocally evaluate the DNA extraction methods and conditions.

**Table 3:** Evaluation of extraction methods and sample mass on heroin samples of varying purities.

Sample	Sample mass (g)	Extraction method	Cycle threshold (C_p_)
WP-1	1.0	Maxwell	32.09
		QP	32.31
		QS	30.89
	2.0	Maxwell	34.38
		QP	33.72
		QS	32.22
BPf-1	1.0	QP	33.10
		QS	29.47
	2.0	QP	34.2
		QS	30.49
BPc-1	1.0	QP	27.64
		QS	29.67
	2.0	QP	28.77
		QS	30.77
NTC	—	—	>35.00
	—	—	>35.00

Lower crossing point (Cp) values indicate higher DNA concentrations. The no-template controls (NTC) are also included and exhibit Cp values > than 35, which indicates an unreliable signal to accurately determine the presence of significant amplification. Note, the Maxwell RNA LEV method was only used to extract DNA from sample WP-1 because the remaining samples lacked the necessary mass to perform the three extraction methods. QP- Qiagen plant Mini kit, QS- Qiagen Stool Mini Kit.

**Figure 2:**
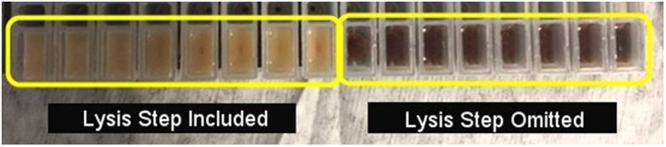
Appearance of white powder heroin samples prepared with the lysis (left) and without lysis (right) using the Maxwell 16 RNA LEV extraction method.

**Figure 3:**
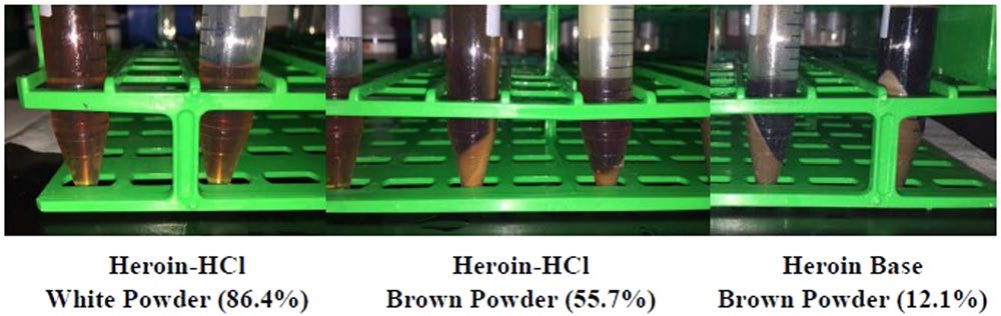
Heroin samples following the addition of P3 buffer (Qiagen Plant Minikit).

Several potentially significant observations were noted during the extractions:

(1) The large mass of the powdered samples (1.0 and 2.0 g samples) required a 2–4 fold increase in the volumes of extraction components. The black tar heroin did not require an increase in the extraction reaction volumes and was instead treated with liquid nitrogen and ground with a mortar and pestle (similar to “gummy” opium). (2) Accommodating the increased sample volumes required the use of several columns (Qiagen) or wells (Maxwell) per sample. Like-samples were then pooled during post-extraction concentration. (3) The use of the Maxwell Tissue RNA LEV lysis procedure had a substantial effect on the appearance and consistency of the sample (Fig. 2). The sample that was not lysed appeared darker and had insoluble components present that could not be removed via centrifugation. The lysed sample went into solution rapidly and had no visible insoluble components. The white powder heroin appeared to go into solution faster than the other samples and consistently had fewer insoluble components removed during the initial preparation of samples for extraction. (4) Following the addition of the Qiagen Plant Mini kit buffer P3 (3.0 M potassium acetate, pH 5.5) and centrifugation, large pellets were obtained, increasing in size as purity decreased (Fig. 3). This step precipitates proteins, large chromosomal DNA molecules, and other containments and will promote DNA adsorption to the silica membrane. There is a high likelihood that “cutting” agents represent a large portion of the precipitate.

qPCR amplification using a poppy specific primer set (N263) was performed to assess the DNA yield and the associated quality of the extraction methods. This indirectly provides a relative assessment of the starting quantity of heroin and poppy DNA in the sample and will allow preliminary comparison of DNA quantity and the heroin purity and type. We adjusted the PCR components and cycling parameters to better contend with the potentially damaged DNA. The 1.0 g and 2.0 g samples yield similar Cp values, with an average difference of 1.34 ± 0.441 cycles. The 1.0 g samples displayed lower Cp values than the 2.0 g samples across all extraction methods, although the 2.0 g samples exhibited higher levels of fluorescence. In practice, we recommend the use of 2.0 g of sample due to the more robust response that was achieved. However, for the purposes of this study, 1.0 g will be used to allow additional comparisons with limiting amounts of samples.

**Figure 4:**
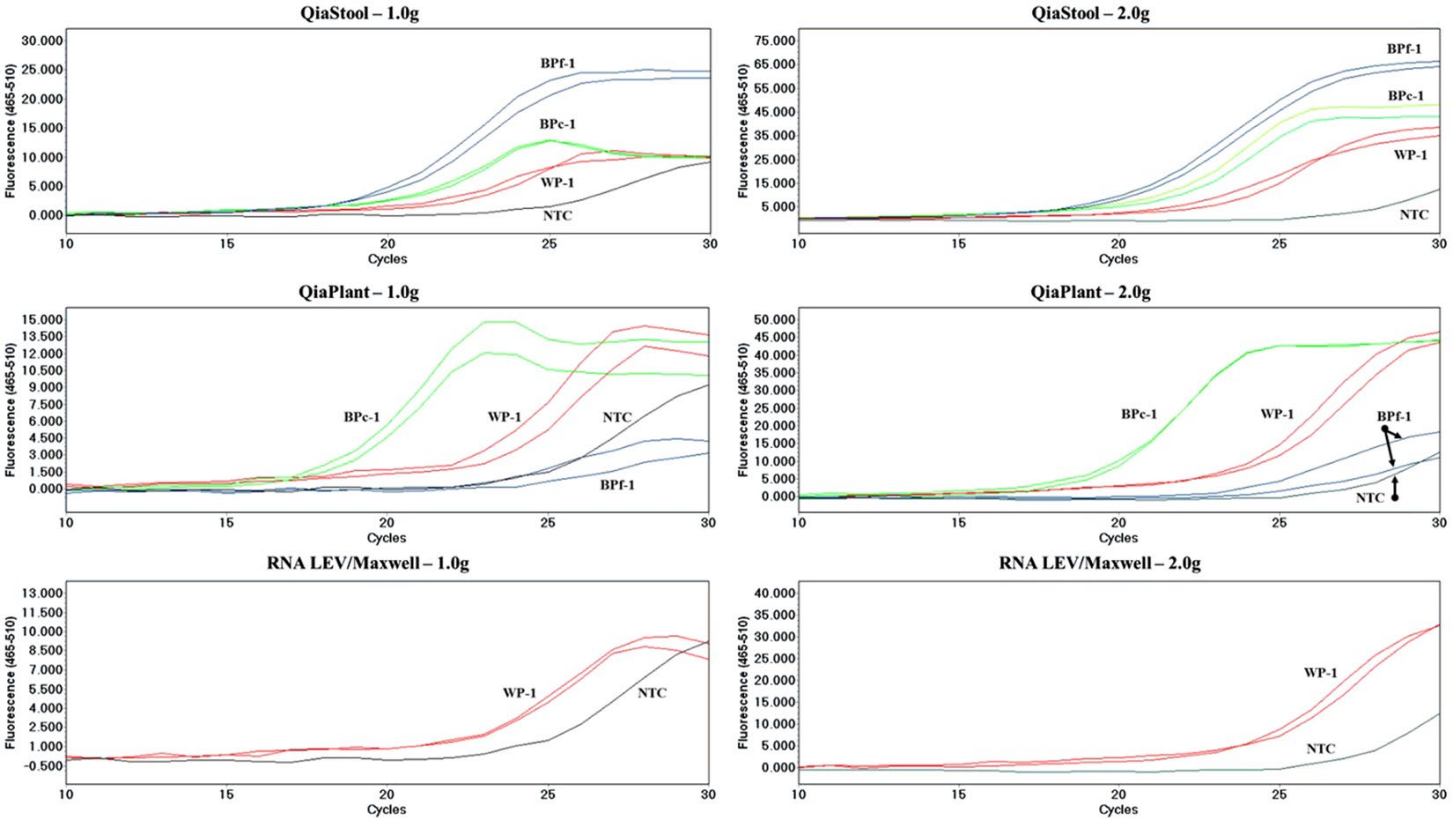
Amplification plots (primer N263) of DNA extracts generated using the QiaStool (top), QiaPlant (middle) and RNA-LEV Maxwell 16 (bottom) extraction methods from 1.0 (left) and 2.0 g (right) of heroin samples – white powder (WP-1), fine brown powder (BPf-1) and coarse brown powder (BPc-1). Note, WP-1 is the only sample with sufficient mass to extract via the RNA-LEV Maxwell 16 method.

**Figure 5:**
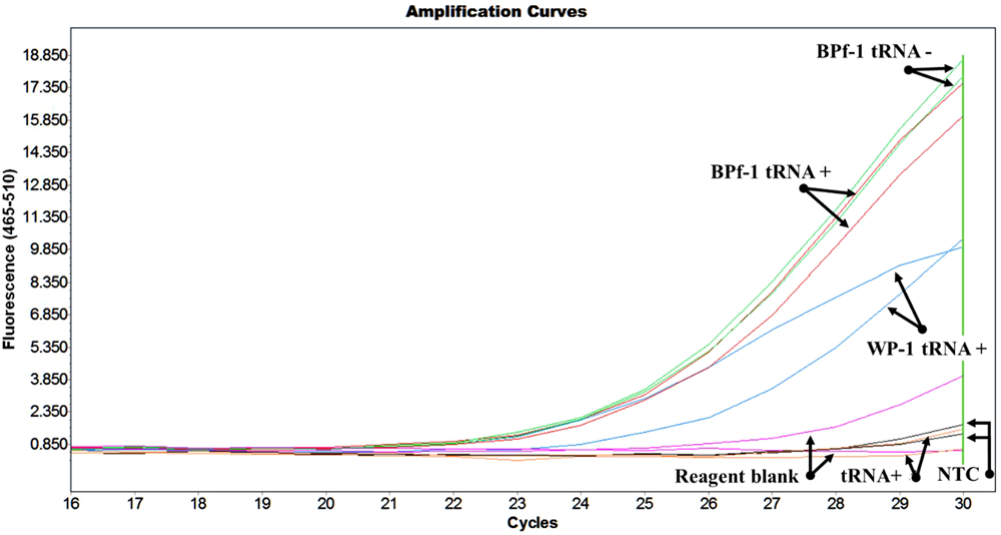
Quantitative PCR amplification plot displaying successful PCR amplification of poppy DNA from heroin samples. The amplification displays the accumulation of double stranded DNA vs cycle, thus the increase in fluorescence at lower cycles indicate higher concentrations of DNA.

**Figure 6:**
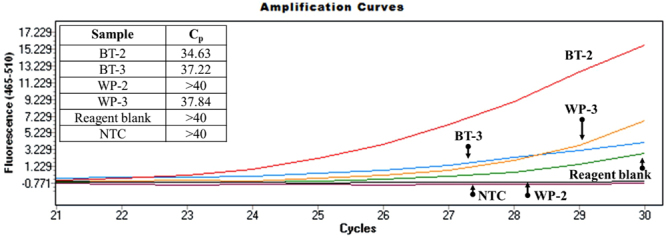
A qPCR amplification curve comparing the responses of DNA amplified from black tar (BT-2 and BT-3) and white powder heroin (WP-2 and WP-3) using primer N263. Note the table in the upper left containing the cycle threshold values (Cp).

The QiaStool DNA extraction kit provided the highest and most consistent DNA yields, with Cp values ranging from 29.47 to 32.22 cycles and an average Cp of 30.59 ± 0.98, while the QiaPlant method resulted in a range of 27.64 to 34.22 cycles and an average Cp of 31.62 ± 2.75 (Fig. 4 and Table 3). The no-template controls displayed Cp values >35.00, which is a late Cp value and unreliable measure of quantity. A late Cp (>35.00 cycles) occurred in only one replicate of the Maxwell extracted WP-1 2.0 g sample; all other samples resulted in interpretable Cp values. The NTC samples across runs had reproducible signals, with melting temperatures between 77.75 and 78.6 °C. Peaks corresponding to this T_m_ range were not obtained in QiaPlant or QiaStool extracted samples (Supplementary Information Fig. 2A). The Maxwell samples did contain a peak in this range; however, additional peaks were detected, indicating additional non-primer dimer/contaminant product was present. This supports the conclusion that the Maxwell extraction method is not the appropriate method for samples of this type. Additional observations are noted in Supplementary Information (Figs 3A–6A).

A second qPCR run was performed using samples that were concentrated and purified using the Microcon YM-30, pre-treated with yeast tRNA (Microcon column^24^), and amplified using the 2-stage quantitative PCR and the N263 primer set. The results from the first run confirmed the successful amplification of poppy DNA from heroin without any background noise from the tRNA (Fig. 5 and Table 4). The BPf-1 heroin sample was amplified in the absence of tRNA, demonstrating that the heroin sample remains amplifiable in the absence of tRNA priming of the Microcon YM-30 column. The no-template control, yeast tRNA, and one replicate of the reagent blank, did not show detectable activity in the amplification plot. The first replicate of the reagent blank had a C_p_ value of 37.63 that may indicate either contamination or background. The heroin samples had C_p_ values of approximately 33.50, detected three cycles prior to the reagent blank.

**Table 4:** Cycle crossing points (Cp) resulting from the amplification of poppy DNA from heroin samples.

Sample	Cp	Avg. Cp
WP-1 tRNA stool	33.16	34.19
	35.22	
BPf-1 tRNA stool	33.61	33.42
	33.23	
BPf-1 stool	33.24	33.20
	33.16	
Reagent blank 120414 stool tRNA	37.63	—
	No activity	
NTC	No activity	—
	No activity	
tRNA	No activity	—

A comparison of commercially available (illustra GenomiPhi v3) and in-house whole-genome amplification methods was performed. We hypothesized that the presence of damaged DNA (lesions, deamination) may hinder the DNA polymerase used in the commercial kit. The in-house method included use of commercially available random hexamers (DNA fragments of six bases) and the 2-step amplification. The study was restricted to two samples due to the limited availability of samples. The results (not shown) indicated that in the current state, neither method improved the quantity of amplifiable DNA.

Successful DNA extraction and subsequent PCR amplification of black tar and brown/white heroin samples were compared using the Syracuse University qPCR procedure described previously. The data further demonstrated that both black tar and powder heroin can be amplified at low levels (Table 5 and Fig. 6). The black tar sample amplified with higher efficiency than the white/brown powder samples (Figs 5 and 6); however, due to the limited nature of this study, it cannot be identified as an expected trend. Black tar is crudely manufactured and therefore the dense, sticky, and gummy sample matrices vary from sample to sample. Despite this challenging matrix, we were able to successfully extract *Papaver somniferum L*. DNA from black tar heroin.

**Table 5:** Heroin samples used to compare the performance of the DNA extraction and 2-stage amplification of black tar and white/brown heroin samples.

Sample	Purity (%)	Description	Cut/Uncut
BT-2	47.8	Black Tar	Uncut
BT-3	50.9	Black Tar	Uncut
WP-2	Unknown	White/off white powder	Unknown
WP-3	88.1	White/off white powder	Uncut

### DNA Isolation- Opium Gum and Latex

Raw opium is produced through the collection and, typically, sun drying of poppy latex. This minimal processing leads to the retention of cellular material and maintains the integrity of the DNA. The challenge for raw opium is in the purification of DNA from inhibiting compounds and the texture of the sample. “Cooked” opium and heroin are further refined through mechanical and chemical methods, decreasing the quantity and quality of DNA present in the sample.

**Figure 7:**
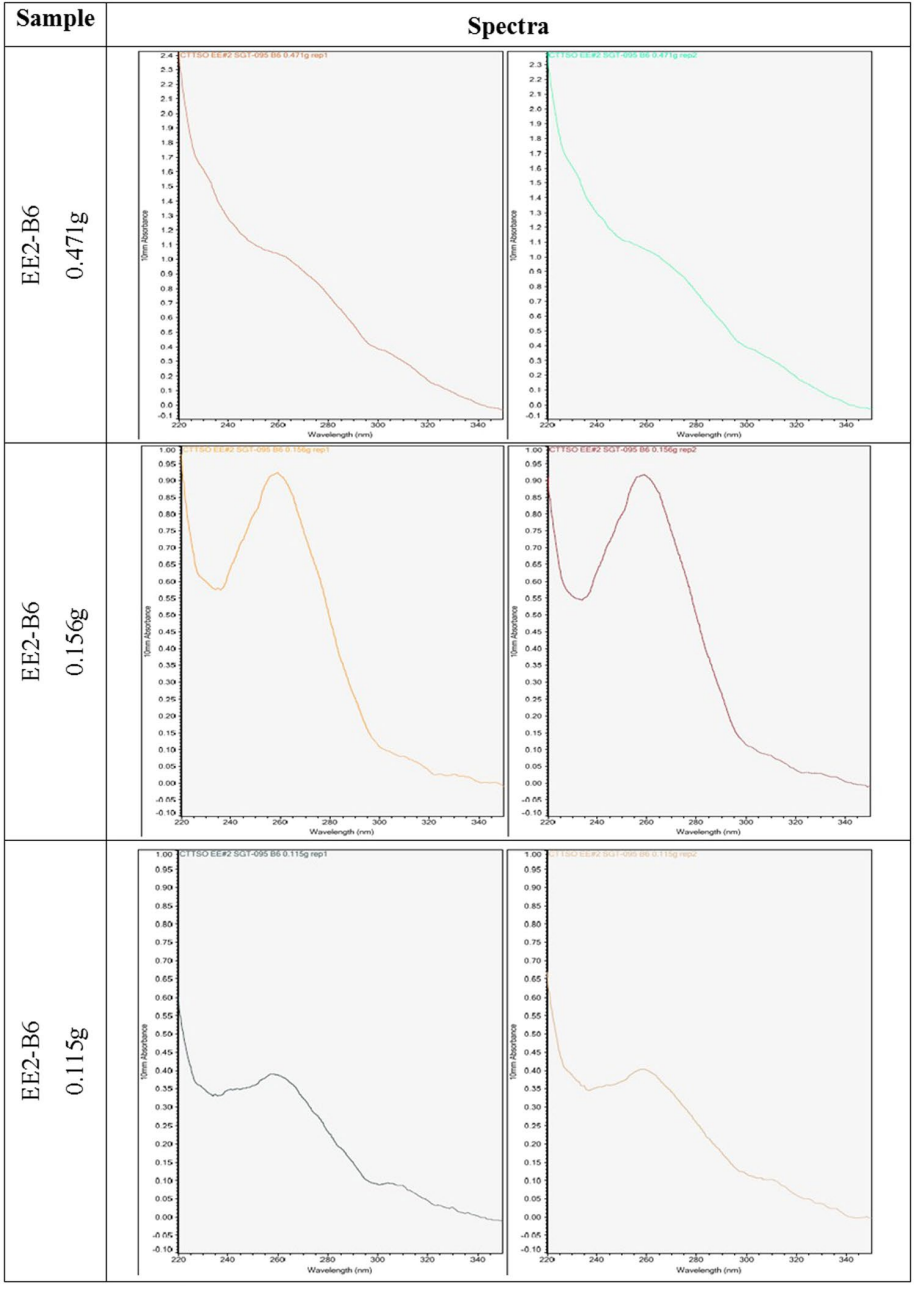
UV absorbance data obtained from Qiagen-based raw opium DNA extractions.

The first experimental objective was to identify the lowest raw opium sample mass that would provide reliable and robust results. Raw opium sample EE2-B6 (Table 6) was extracted in triplicate using Qiagen Plant DNeasy DNA extraction kit^25^ using sample masses of 0.471 g, 0.156 g and 0.115 g, respectively. The absorbance data obtained from the spectrophotometer provides an indication of sample purity, and thus indicates potential PCR performance. The results indicate that the 0.156 g sample exhibited the most favorable overall yield and sample quality, with an average 260:280 ratio of 1.86 and a 260:230 ratio of 1.59 (Table 6 and Fig. 7). DNA extractions from opium will target ~0.15 g of starting material. The target mass may vary due to the multitude of different characteristics displayed in an illicit opium sample, *e.g*., the varying densities and/or concentrations of samples may require an increase or decrease in sample mass to achieve similar amounts of DNA. The differences in quality of the material (dried, gummy or cooked) will likely play the most significant role in determining the amount of starting material (Supplementary Information: Fig. 1A). A sample that is overly desiccated and exposed to high temperatures (cooked) will undoubtedly require an increase in the amount of starting material.

**Table 6:** Absorbance-based nucleic acid quantitation (Nanodrop) from EE2-B6 raw opium.

Sample	Mass (g)	Replicate	ng/µL	260:280	260:230
Raw opium EE2-B6	0.471	1	51.4	1.38	0.64
		2	51.9	1.38	0.65
	0.156	1	45.7	1.88	1.53
		2	45.5	1.83	1.64
	0.115	1	19.2	1.67	1.10
		2	19.9	1.57	1.04

The second study focused on identifying an efficient DNA extraction method that results in an acceptable yield and purity of DNA. The extraction method evaluated include: (1) an automated Promega Maxwell 16 Tissue RNA LEV silica/magnetic bead-based method^23^ and (2) the Qiagen Plant DNeasy silica column-based DNA extraction method. Nucleic acids were successfully extracted from all attempted raw opium samples; the Promega Maxwell 16 RNA LEV method was successful in all 15 samples while the Qiagen Plant DNeasy yielded detectable DNA in nine of 15 samples (Table 7). The Qiagen-based method will likely result in an increasingly pure extract when compared to the Promega method, with 260:280 values of 1.44 ± 0.64 and 1.25 ± 0.31, respectively. Sample EE3-2, extracted using the Qiagen method, exhibited a poor yield (−6.5 ng/µL; Table 7- shaded cell); this is likely an anomaly and is considered an outlier. The average 260:280 ratio for the Qiagen-extracted samples improved to 1.61 ± 0.43 when this sample was removed.

**Table 7:** Raw Opium DNA yields (ng/µL) and 260:280 nm ratios.

Sample ID	Promega (final volume ~45 µL)		Qiagen (final volume ~70 µL)	
	Nucleic Acid (ng/µL)	Avg. 260/280	Nucleic Acid (ng/µL)	Avg. 260/280
EE2-B1	44.55	1.785	—	—
EE2-B2	21.05	1.28	8.798	1.33
EE2-B3	13.75	1.25	10.943	1.964
EE2-B4	27.85	1.47	10.362	2.0775
EE2-B5	3.2	1.675	67.406	2.26
EE2-3	25.2	1.875	—	—
EE2–4	10.3	1.855	—	—
EE2–5	8.05	1.29	3.469	1.472
EE3-1	12	1.46	14.412	1.4175
EE3-2	3.5	1.245	*−6.5305*	0.102
EE3-3	0.6	0.57	0.8625	1.15
EE3-4	3.1	1.05	20.3955	1.188
EE3-5	13.2	1.735	—	—
EE3-6	60	1.375	—	—
EE3-7	8.95	1.67	—	—

The italicized value represents an outlier and was effectively removed from the final analyses.

The higher moisture content of the opium samples had a negative impact on the quality of results. DNA extractions were, in general, less successful when the opium samples were “gummy”. The inability to increase the sample’s surface area during bead beating may have caused this phenomenon. To mitigate this problem we attempted two approaches: pretreatment with liquid nitrogen and air-drying. We found that in isolation, the air drying/bead-beating far outperformed the treatment of liquid nitrogen followed by bead-beating (data not shown). The preferred sample pre-processing method included air-drying in a fume hood and, as needed, treatment of the sample with liquid nitrogen followed by physical disruption with a mortar and pestle and bead-beating. This greatly increased the surface area of the opium sample exposed to the lysis buffers while simultaneously decreasing the activity of any endogenous DNA degrading DNases.

We recommend extracting poppy DNA from 0.15 g of raw opium using the Maxwell 16 RNA-LEV method followed by concentration/purification using DNA Fast Flow Microcon centrifugal filtration devices (EMD Millipore Corporation). The sample quality was more advantageous in the Qiagen extraction method; however, it is also significantly more labor intensive and, when considering the 260:280 ratio standard deviations, the two methods perform similarly. The DNA isolation method developed for latex is similar to the method developed for opium. Latex samples are commonly resuspended in water for long-term storage; therefore, the opium extraction method was modified to include the concentration of biological material. Latex samples had a lower DNA yield and low quality values (Table 8).

**Table 8:** Nucleic acid and purity metrics obtained from latex samples extracted using the modified Maxwell 16 RNA-LEV DNA extraction method.

Latex Sample	Mean [nucleic acid]	Mean 260/280	Mean 260/230
1	13.65 ± 1.061	1.48 ± 0.057	0.51 ± 0.014
2	5.55 ± 0.071	1.79 ± 0.226	0.41 ± 0.014
3	6.45 ± 0.354	1.885 ± 0.163	0.575 ± 0.007
4	53.2 ± 0.424	1.575 ± 0.021	0.695 ± 0.007
5	20.45 ± 0.354	1.67 ± 0.028	0.6 ± 0
6	8.15 ± 0.354	1.99 ± 0.085	0.61 ± 0.014
7	7.75 ± 0.354	1.75 ± 0.085	0.47 ± 0.014
8	49.2 ± 0.283	1.735 ± 0.021	1.015 ± 0.007
9	11.1 ± 0	1.87 ± 0.057	0.83 ± 0
10	38.45 ± 0.636	1.775 ± 0.021	1.245 ± 0.007
11	41.6 ± 0.141	1.775 ± 0.007	1.205 ± 0.021
12	35.4 ± 0.141	1.81 ± 0.014	1.375 ± 0.021
13	12.5 ± 0.424	1.63 ± 0.014	0.48 ± 0.014
14	41.35 ± 0.071	1.755 ± 0.021	1.265 ± 0.021
15	49.25 ± 0.354	1.735 ± 0.035	1.145 ± 0.021
Reagent Blank	2.45 ± 0.071	1.63 ± 0.113	0.275 ± 0.007

**Figure 8:**
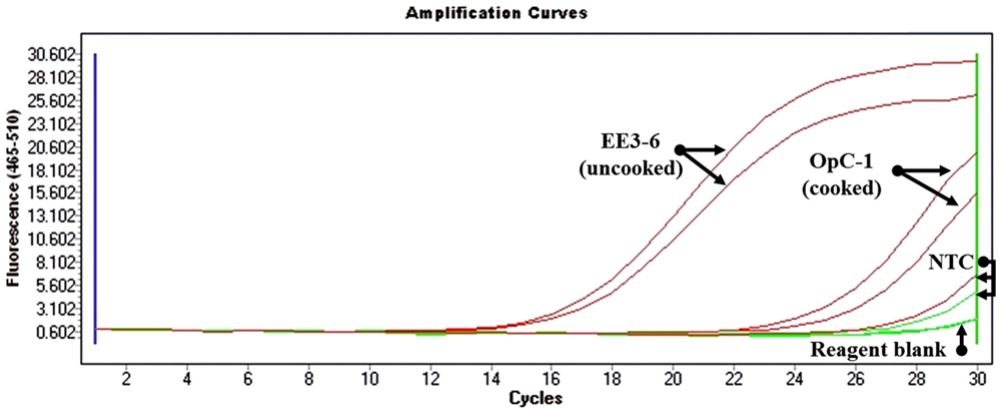
Quantitative PCR (qPCR) amplification plot displaying successful PCR amplification of poppy DNA isolated from both cooked (OpC-1) and uncooked (EE3-6) opium samples.

Opium gum (cooked and uncooked) extractions were evaluated using the new heroin workflow (Qiastool DNA extraction, Microcon concentration with tRNA and 2-stage PCR). Like heroin, we expect this workflow to yield high amounts of amplifiable template DNA. One gram of samples EE3-6 (uncooked) and OpC-1 (cooked) were extracted with the Qiastool kit; InhibitEX buffer and lysis reaction volumes were doubled to ensure samples were properly lysed and washed. Quantitative PCR was performed using the 2-stage amplification and the N263 primer set. The results indicate that DNA originating from both cooked and uncooked opium have been successfully extracted using the Qiastool kit and amplified using the 2-stage amplification method (Fig. 8). The uncooked opium (EE3-6), as expected, had a higher DNA yield, exhibiting an average Cp of 25.48. In contrast, the cooked opium (OpC-1) had a low yield exhibiting a Cp of 34.33 (Table 9). Although both cooked and uncooked opium were successfully amplified, the DNA yield of the cooked opium may be improved through the extraction of higher amounts of starting material; therefore, the current optimal mass of starting material for extractions from cooked opium will be 1.5 g.

**Table 9:** Quantification Cycle crossing points (Cp) resulting from the replicate amplification of poppy DNA of both cooked (OpC-1) and uncooked (EE3-6) opium samples using the N263 poppy primer set.

Name	Cp	Avg. Cp
OpC-1	33.79	34.33
	34.86	
EE3-6	25.71	25.48
	25.24	
Reagent blank 122214GL	No activity	—
	No activity	
NTC	No activity	—

**Figure 9:**
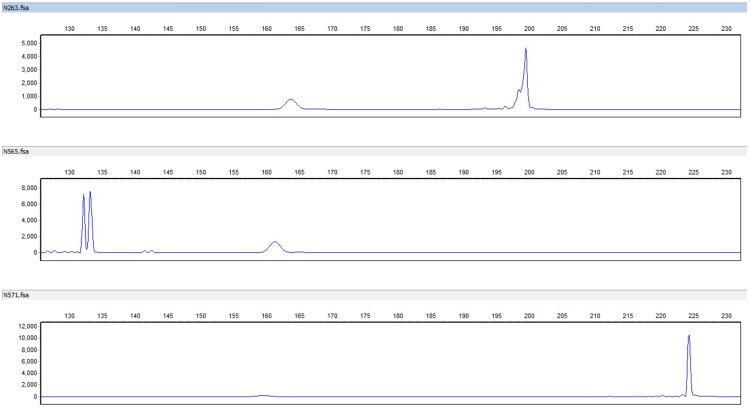
Select electropherograms obtained from the PCR amplification and subsequent CE-analysis of raw opium samples (top-primer N263, middle-primer N565, bottom-primer N571). X-axis is fragment size in bases and Y-axis is relative fluorescent units. Note, a free-dye artifact is present at approximately 160 bases; this is independent of the sample and can be disregarded from analyses.

The extracts were then amplified using fluorescein-labeled poppy specific primers N263, N565 and N571 and analyzed on an Applied Biosystems 3500xL Genetic Analyzer (ThermoFisher Scientific Inc.) capillary electrophoresis (CE) instrument (Fig. 9). The N263, N565 and N571 loci contain microsatellite sequences; therefore it is possible that size polymorphisms exist within the two genomic copies of the DNA in the diploid genome of a poppy plant. Opium sample EE-2 amplified with N263 and N571 yielded a single primary allelic peak (homozygote), with an amplicon length of 199.5 and 224 bases, respectively. The amplification using primer N565 yielded two primary allelic peaks (heterozygote) with amplicon sizes of 132 and 133 bases. The resulting electropherograms also exhibit minimal artifactual noise, such as non-specific allelic peaks caused by electrical spikes or –A peaks caused by the non-template dependent terminal adenylyl-transferase activity inherent to many DNA polymerases, including the Roche Fast Start High Fidelity DNA polymerase. The additional peaks present represent free dye artifacts (“dye blobs”) at 160 bases and “stutter” products generated by polymerase slippage during the amplification of microsatellites. The stutter products correspond to products that are one or two repeat units smaller than the allelic peak. For example, the N571 locus contains a tetra-nucleotide repeat ([TCAT]_n_) which, when amplified, resulted in the expected allelic peak at 224 bases (peak height:10523 rfu) and a minor stutter peak at 220 bases (peak height: 210 rfu). The results demonstrate that Promega-isolated opium samples are of sufficient quality and quantity to yield amplifiable DNA and interpretable results.

### PCR Optimization

**Figure 10:**
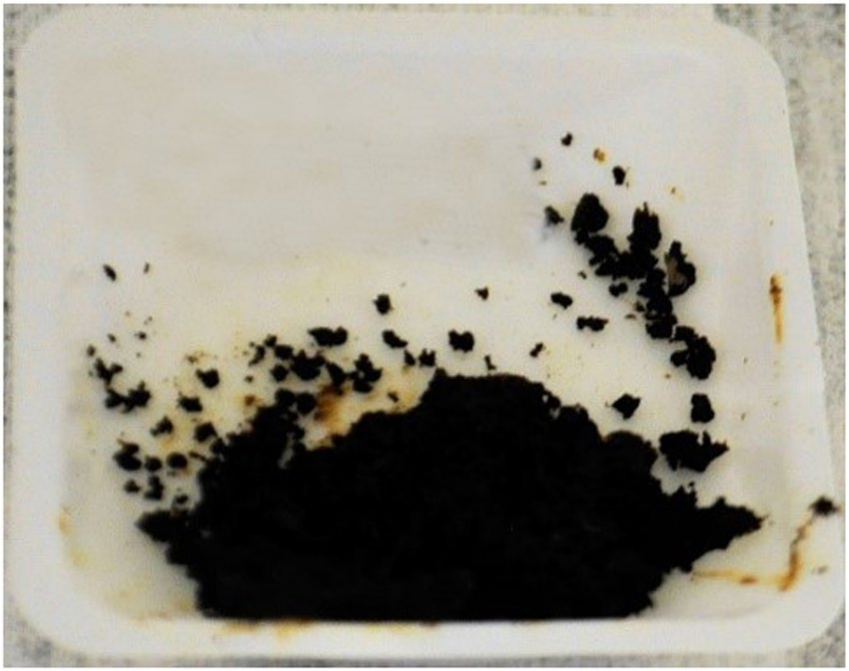
EE3-6 opium sample.

PCR conditions were optimized in anticipation of the low quality and quantity of DNA encountered in processed opium and heroin samples. DNA extracts (Maxwell RNA-LEV method) obtained from the EE3-6 opium and a poppy leaf samples were amplified with three poppy specific assays (N263, N565 and N571) using Omni KlenTaq and Roche FastStart High Fidelity DNA polymerases and PCR buffers with a pH of 7.9, 9.2 and 9.0. The EE3-6 opium sample was anticipated to exhibit elevated levels of inhibition and low DNA quantities due to characteristics such as moisture level, color and texture (Fig. 10).

The combined enzyme master mix with a PCR buffer pH of 9.2 led to successful amplifications across all samples and provided the most consistent results (Table 10). The leaf sample displayed consistent amplification (C_p_) within each assay group. This was expected and served as a benchmark for the lower quality EE3-6 opium sample.

**Table 10:** Cycle Threshold (Cp) values obtained from the amplification of DNA extracts from EE3-6 (opium) and a poppy leaf using three poppy specific assays, varying PCR buffer pH and polymerases.

Assay	pH	Polymerase	EE3-6 C_p_	Leaf C_p_
N263	7.9	combined	35	22.71
		Omni	—	22.96
	9.2	combined	32.7	22.05
		Omni	32.87	21.42
	9	Roche	—	20.76
N565	7.9	combined	—	25.52
		Omni	—	23.72
	9.2	combined	33.37	26.81
		Omni	28.54	23.61
	9	Roche	—	25.51
N571	7.9	combined	35	26.73
		Omni	—	24.57
	9.2	combined	35	26.48
		Omni	—	24.92
	9	Roche	—	24.59

## Discussion

To the best of our knowledge, this paper is the first to demonstrate successful DNA extraction of opium poppy DNA from heroin samples. The primary challenges were the low quality and quantities of DNA expected in processed opium and heroin samples coupled with the challenges posed from sample matrix issues and cutting agents. In that regard, the successful extraction of DNA fragments is very significant. The quantities, as measured through the qPCR crossing point values, were low; however, this was not unexpected. The most critical finding was that we were able clearly distinguish opium poppy amplification plots from negative controls and background noise. Preliminary data indicate that we have successfully sequenced poppy DNA for some of these samples (data not shown).

The opium (gum or latex) contains a mixture of alkaloids, terpenoids, phenols, and various proteins^26^. Opium is simply the dried latex that is collected from the opium poppy. It is typically in the raw form (only dried) or in the cooked form (where raw opium is boiled, filtered to remove impurities, and reconstituted) for heroin production. The development of a successful DNA isolation strategy relies heavily on the ability to address the complexities present in latex or opium gum samples. This includes the presence of compounds known to inhibit PCR such as phenolics and polysaccharides and the “gummy” texture of opium samples.

We confirmed that standard plant DNA extraction kits would be sufficient to extract poppy DNA from opium. Despite the increased quality metrics, the Qiagen procedure remains more labor intensive and less reliable compared to the automated Promega methods. Despite this, we recommend the use of the Qiagen stool kit as it proved to be more versatile. The stool kit has been optimized for low quantity/quality samples that contain high level of PCR inhibitors and is more appropriate for the wide variety of opium and heroin samples that we encountered.

In order to improve the DNA yield and overall quality of heroin samples further, we employed a 2-stage amplification reaction using two DNA polymerases. This dual polymerase system attempts to balance the amplification of damaged DNA while maintaining a high fidelity and minimize errors. The Omni KlenTaq DNA polymerase (DNA Polymerase Technology) is highly resistant to inhibitors and DNA lesions^27–29^ while the Roche Fast Start/High Fidelity DNA polymerase (Roche Diagnostics) is more susceptible to inhibitors and DNA lesions but is highly processive and will aide in minimizing amplification errors. The 2-stage system was performed in two successive cycles, a 10-cycle reaction (PCR-1) using the Omni KlenTaq polymerase followed by a 30-cycle reaction (PCR-2) with the Roche FastStart high fidelity polymerase. PCR-1 amplifies the DNA extract with the Omni KlenTaq DNA polymerase (DNA Polymerase Technology). This polymerase is highly efficient in the presence of PCR inhibitors and damaged DNA, both of which are expected in heroin and opium DNA extracts. The Omni KlenTaq DNA polymerase, however, has an elevated error rate (0.027% insertions, 0.0035% deletions 0.2369% substitutions)^27^. PCR-2, using the Roche FastStart High Fidelity Taq DNA polymerase, was used to obviate the elevated error rates and absence of proofreading exonuclease activity with Omni Klentaq^30^. The FastStart High Fidelity DNA polymerase exhibits exceedingly low error rate (4 × 10^−6^)^31,32^. This method led to more consistent responses than either polymerase alone.

The combined effects of exceedingly low levels of damaged template DNA and the presence of inhibitors necessitated the modification of conventional analytical methods to increase sensitivity, for example, the 40-cycle, 2-stage amplification mentioned above. The combined use of these methods resulted in the successful detection of opium poppy DNA from heroin and opium samples, but it also may have contributed to the low-level activity observed in the no-template controls. The most commonly detected non-specific signals are due to the formation of primer dimers. The protocol was initially optimized using a 300 nM primer concentration and a lead extract rather than an extract from a heroin sample. The primer concentration can be lowered to avoid dimerization. In addition, the use of such a high concentration is likely unnecessary when amplifying low-copy samples like those obtained from heroin extracts. The Cp values for the NTCs were greater than 35 cycles and were identified by the Roche LightCycler 480 software as unreliable due to high Cp values. The activity of the NTC is on average 4.4 and 3.4 cycles greater than the QiaStool and QiaPlant extracted samples, respectively (Fig. 4 and Table 3). The heroin-positive samples exhibited T_m_ (s) distinct from the activity detected in the NTC with clear differences in curve morphology (Supplementary Information Fig. 2A). In addition, the signal obtained from NTC samples are reproducible across amplification reactions and instrument runs. We conclude that the NTC activity is not responsible for the primary signal obtained from the heroin positive samples.

While we believe that our proposed methods perform sufficiently well with high purity heroin samples (i.e., no cutting compounds), further validation needs to be performed using heroin samples containing varying amounts of adulterants or diluents. Our inventory of opium and heroin samples included over 200 samples consisting of white and brown powder and black tar heroin, as well as several types of opium. However, these samples were also limited in quantity due to the nature of the seizures in source countries. In addition, few samples had ample mass to enable replicative comparisons of the methods we evaluated in this study. This limited the depth of our sub-studies and we recommend that the data presented herein be used as a starting point for further refinement of the procedure to extract opium poppy DNA from heroin. For example, our small whole genome amplification sub-study indicated that there was no benefit to the use of these methods. We believe that given additional samples for amplification of the whole genome would increase the amount of “template DNA” and improve subsequent genotype analyses. Due to the nature and type of samples, any research group would face similar limitations to successful identification of poppy DNA caused by sample availability.

The method described in this paper represents the first time DNA from the opium poppy (*Papaver Somniferum L*.) has been successfully isolated from heroin samples. The ability to obtain an opium poppy DNA profile from heroin opens a host of new avenues for law enforcement. This genetic information, through the use of high throughput sequencing, will likely enable the identification of the source country or region of seized heroin and positive identification of poppy-derived illicit drugs. This lays the foundation for the information that can then be used for interdiction of the drug trade from a local to a global scale. Source information may provide valuable intelligence leading to the interruption of this terror-funding stream or other illegal activities.

## Methods

QIAamp Fast DNA stool mini kits were purchased from Qiagen (Hilden, Germany). Microcon Ultracel YM-30, Microcon DNA Fast Flow, and Proteinase K Tritirachium album were purchased from EMD Millipore (Billerica, MA). Maxwell 16 Tissue LEV RNA purification kits were purchased from Promega (Madison, WI). DNA LoBind Tubes, both 1.5 mL and 5.0 mL, were purchased from Eppendorf (Hauppauge, NY). Omni KlenTaq was purchased from DNA Polymerase Technologies (St. Louis, MO). Fast Start High Fidelity PCR System, dNTP packs were purchased from Roche Life Science (Indianapolis, IN). Bovine Serum Albumin (20 mg/mL) was purchased from New England Biolabs (Ipswich, MA). Transfer Ribonucleic Acid (tRNA) from baker’s yeast (*S. cerevisiae*) and absolute ethanol were purchased from Sigma Chemical Company (St. Louis, MO). Poppy, opium and heroin samples were primarily provided by DEA Special Testing and Research Laboratory.

Several DNA extraction methods were evaluated during the course of this study: (1) the Qiagen Plant DNeasy Mini kit, (2) the Promega Maxwell 16 Tissue RNA LEV kit and (3) a modified version of the Qiagen Stool kit DNA extraction method. DNA extraction negative controls (reagent blanks or RB) were included in all DNA extraction sets. The reagent blanks consist of all reagents used in the extraction and without added DNA and were processed and analyzed (quantified and amplified) in concert with all experimental samples.

### DNA extraction from opium gum and latex

DNA was extracted from opium gum by first mechanically pulverizing via mortar and pestle, either 1.0 g of dried or cooked samples, or 1.5 g of sample when the samples had a “gummy” texture. “Gummy” samples were pretreated with liquid nitrogen prior to grinding; the resulting powder was placed in a 5 mL Eppendorf LoBind tube with 2 mL of InhibitEX buffer and incubated for 10 minutes in a 65 °C water bath. After the incubation, 50 µL of Proteinase K and 1200 µL of Qiagen Buffer AL were added to the tube and vortexed before returning to the 65 °C water bath for 2 hours. After incubation, the samples were centrifuged at 4150 × g for 1 minute and 1200 µL of supernatant were removed and placed in a new 5 mL LoBind tube along with 1200 µL of absolute ethanol. Samples were loaded into QIAamp columns and extracted as per the QIAamp extraction protocol and eluted into 100 µL of ATE buffer. The samples were stored at 4 °C for short term or −20 °C for long term before concentration occurred.

Latex samples are typically suspended in water; therefore, the extraction technique in this method was similar to that used for opium, but it required removal of the aqueous phase. The water was removed from 1 mL of each sample through centrifuging at 14300 × g for 2 minutes, removing the aqueous layer, spinning again at 1600 × g for 2 minutes and removing any remaining water. The sample was then disrupted by vortexing for 2–6 minutes using zirconia and glass beads of varying diameters in a tissue disruptor (bead beater). The remaining procedure is identical to the opium procedure.

### DNA extraction from Black Tar Heroin

Black tar heroin is particularly difficult to handle due to the gummy yet dense nature of the sample. DNA was extracted from black tar heroin by first freezing the entire sample vial at −20 °C for at least 10 min. The samples were then flash frozen in liquid nitrogen, scraped within the vial. The frozen material was broken up in the vial and scraped from the sides into a powder and 2.0 mL of InhibitEX buffer was added directly to the vial. This was incubated in a 70 °C water bath for 20 minutes. The vial was vortexed every 5 minutes to encourage the sample to go into solution. After the incubation, 50 µL of Proteinase K and 1200 µL of Qiagen Buffer AL were added to the vial and the solution was mixed before returning to the 70 °C water bath for 2 hours. The samples were transferred to 5 mL Eppendorf LoBind tubes and centrifuged at 4150 × g for 1 minute and 1200 µL of supernatant were removed and placed in a new 5 mL LoBind tube along with 1200 µL of absolute ethanol. Samples were loaded into QIAamp columns and extracted as per the QIAamp extraction protocol and eluted twice with 50 µL of ATE buffer

### DNA extraction from Powdered Heroin

DNA was extracted from powdered heroin by combining 2 mL of InhibitEx buffer with approximately 1.0 g of sample in a 5 mL Eppendorf LoBind tube. If a dry pellet formed, vortexing was needed. The sample was then incubated in a 70 °C water bath for at least 10 minutes with frequent vortexing to ensure the material went into solution. The sample was then centrifuged at 4150 × g to pellet any insoluble materials and 1200 µL of supernatant were transferred to a new 5 mL tube. 50 µL of Proteinase K and 1200 µL of Qiagen Buffer AL were added to the transferred supernatant and mixed before returning to the 70 °C water bath for 2 hours. The samples were then centrifuged at 4150 × g for 1 minute and 1200 µL of supernatant was removed and placed in a new 5 mL Eppendorf LoBind tube along with 1200 µL of absolute ethanol. Samples were loaded into QIAamp columns and extracted per the QIAamp extraction protocol and eluted into 50 µL of ATE buffer.

### Post Extraction Concentration/Purification

In order to maximize amplification of low copy number samples, DNA extracts were concentrated prior to amplification. Using Microcon YM-30 (heroin samples) or DNA Fast Flow (latex or opium samples) concentrators, columns were pretreated with 200 µL of yeast tRNA (5ug/mL) and samples were centrifuged at 500 × g for at least 12 minutes, or until the majority of the buffer or water was spun through the column however avoiding spinning to dryness. A second wash was performed if the filtrate was not clear; 400 µL of deionized water was added to the column and centrifuged at 500 × g for 12 minutes. After removal of the filtrate, 10–20 µL of deionized water or Tris-EDTA, pH8.0 (TE) was added to the membrane, bringing the total volume of liquid on the membrane to ~25 µL. The filtrate column was inverted in a new Microcon tube before spinning at 700 × g for 3 minutes.

DNA extracts (2.0 µL) were quantified in duplicate using the NanoDrop 2000 (Thermo Fisher Scientific Inc.) and the Biotek Synergy HT spectrophotometer (Biotek). All DNA amplifications used a Roche LightCycler 480 II. Amplification of the samples was carried out with a two-step cycling method using two different polymerases. The first round of amplification used the Omni KlenTaq polymerase (Tables 11 and 12). This was used to combat the low quality of the DNA expected in opium and heroin samples. The Omni KlenTaq master mix was spiked with a 1:25 dilution of the Roche Fast Start High Fidelity polymerase to take advantage of the proofreading activity. The second round of amplification was carried out using Roche Fast Start High Fidelity polymerase without purification of the sample (Tables 13 and 14). qPCR was performed on a Roche Light Cycler 480 using SYTO-9 (Thermo Fisher Scientific Inc.), a green fluorescent intercalating nucleic acid dye. The fluorescence crossing point (C_p_) generated in the LightCycler 480 software 1.5.0 was used to compare amplifications across different samples. Controls were included in every quantitation and amplification. DNA extraction negative controls (reagent blanks or RB) consisting of all reagents used in the extraction and without added DNA were processed and analyzed (quantified and amplified) with samples with added material. PCR negative controls (No-template controls, NTCs) consisting of the amplification reagents and specific primer sets used within each reaction were included with every qPCR run. Reagents blanks and NTCs did not demonstrate significant activity in any qPCR runs. This indicates that the extractions were contaminate free and the primers did have aberrant specificity for any DNAs that may be present in the samples that were not poppy DNA.

**Table 11:** Omni KlenTaq master mix components.

**#**	Reaction Component	Volume (µL)
1	10× PCR Buffer pH 9.2	1.5
2	dNTPs	0.36
3	Omni KlenTaq	0.09
4	BSA	0.6
5	Primer	1.05
6	dH2O	1.4
7	Sample	10

**Table 12:** Omni KlenTaq master mix cycling parameters.

Step		Temperature °C	Time
Initial Denature		94 °C	4 m
10 Cycles	Denature	94 °C	30 s
	Anneal	Target Specific	45 s
	Extension	72 °C	60 s
Termination		72 °C	10 m

**Table 13:** Roche Fast Start High Fidelity master mix comments.

**#**	Reaction Component	Volume (µL)
1	10× PCR Buffer w/o MgCl_2_	1.5
2	dNTPs	0.05
3	Roche Taq	0.11
4	BSA	0.2
5	Primer	0.35
6	dH2O	to 15 µL
7	Sample	10

**Table 14:** Roche Fast Start High Fidelity master mix cycling parameters.

Step		Temperature °C	Time
Initial Denature		95 °C	15 m
30 Cycles	Denature	95 °C	30 s
	Anneal	Target Specific	45 s
	Extension	60 s	60 s
Termination		72 °C	5 m

Amplicons were purified using AMPure XP beads following the standard protocol with a 1.8 to 1 ratio of beads to sample. Alternatively, gel purification could be done in place of the AMPure procedure.

### DNA marker specificity and primer design

The DNA markers targeted in this study (N263, N565 and N571) were previously mined from genomic data obtained from over 25 *Papaver somniferum L*. individuals. The markers contain microsatellite sequences for use in potential downstream genetic identification assays (Supplementary Information: Table 13). The marker sequences were queried using the NCBI BLAST database^33^. MegaBLAST^34^ results did not return any hits, while Blastn results yielded hits with a maximum query coverage of 26% and no e-value under 0.088, with the majority over 1.0. These results indicate that these sequences are unique. In addition, the primerBLAST query results did not return any target sequences within the nr database, therefore specificity for the *Papaver somniferum L*. targets are expected^35^. Note, the poppy-specific primer sequences used in this study are available from the corresponding author on reasonable request.

Capillary electrophoresis was used to further characterize the DNA markers. 2.0 µL of each DNA extract was amplified using the parameters discussed in previous sections; 1 µL of each amplicon was combined with 9 µL of a formamide-GS600LIZ size standard master mix and analyzed on an Applied Biosystems 3500xL Genetic Analyzer (Thermo Fisher Scientific Inc.). Capillary electrophoresis was performed using an injection voltage of 1.6 kV and a 10 s injection time.

### Data availability

The data relating to poppy-specific primer sequences used in this study are available from the corresponding author upon reasonable request.

## Electronic supplementary material

Supplementary information
