# Supplementary material for: Development of a Method to Extract Opium Poppy (Papaver somniferum L.) DNA from Heroin
Source: Sci Rep. 2018 Feb 7;8:2590. doi: 10.1038/s41598-018-20996-9 (PMC5803222; doi:10.1038/s41598-018-20996-9)
Supplement: Supplementary file 1 — Supplementary information [file 41598_2018_20996_MOESM1_ESM.doc]

Development of a Method to Extract Opium Poppy (*Papaver somniferum L.*) DNA from Heroin

Authors: Michael A. Marciano, M.S.^1*^, Sini X. Panicker, M.S.^2^, Garrett D. Liddil, M.S.^1^, Danielle Lindgren, M.S.^1^, Kevin S. Sweder PhD.^1^

^1^ Forensic & National Security Sciences Institute, Syracuse University, Syracuse, New York 13244 USA

^2^ U.S. Drug Enforcement Administration, Special Testing and Research Laboratory, Dulles, VA 20166 USA.

*Corresponding author, email: mamarcia@syr.edu

**Supplementary Information**

Table 1: Opium Poppy microsatellite marker repeat structure. Note, “X” represents any base, A,C,T or G.

| Microsatellite Marker | Repeat structure |
| --- | --- |
| **N263** | (CAA)_n_ |
| **N329** | (CAG)_n_(CAA)_n_X_n_ (CAG)_n_ X_n_(CAA)_n_X_n_(CTG)_n_X_n_(CAA)_n_(CAG)_n_(CAA)_n_X_n_(CAA)nX_n_(CAG)_n_ |
| **N565** | (CCA)C(CTT)(CCA) |
| **N570** | (AGGTT)_n_ |
| **N571** | (TCAT)_n_ |
| **Nt513** | (TCTT)_n_ |
| **Nt537** | (CAGAG) _n_ CAGG(CAGAG)_n_ |

Figure 1A: To determine the level of moisture still present in opium samples, a scale from 1 to 5 was created (top - 1 through 4 left to right; bottom-5). Consistency was measured using pipette tips. Scale: 1‐ nearly fluid with molasses-like stickiness; 2‐ high moisture but less easily manipulated; 3‐ maintained solid form but could be shaped under slight pressure.; 4‐ low in moisture but still able to be pierced; 5‐ very dry, brittle and unable to be pierced.


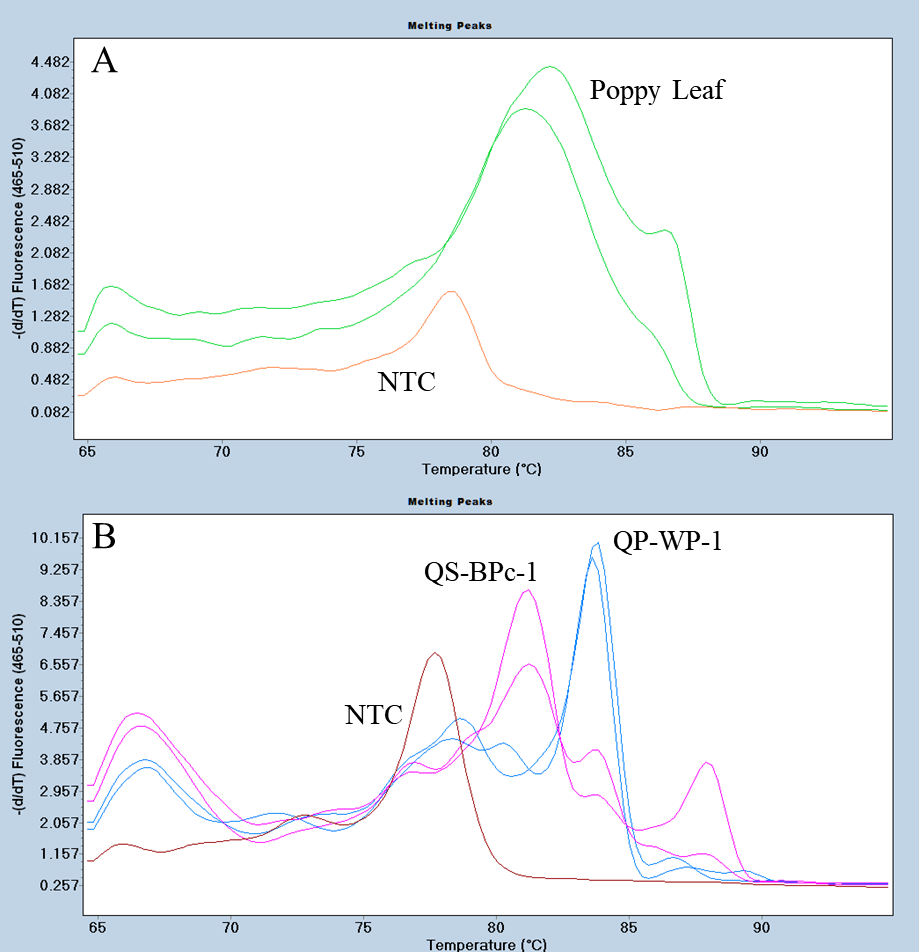


Figure 2A: Melting Curve comparison of signals obtained from amplification using primer N263 and (A) a QiaPlant extracted poppy leaf sample and NTC and (B) Qiastool extracted coarse brown powder heroin –HCl (QS-BPc-1), QiaPlant extracted white powder heroin (QP-WP-1) and NTC. The NTC activity is consistent across the separate runs and extractions. This demonstrates the NTC activity present did not preclude the conclusion that DNA originating from a poppy plant is present. Note, the poppy (or heroin) positive samples do not have reproducible melt curves. Heroin samples contain DNA originating from an unknown number of individual poppy plants, which will be sample-specific in many cases. This variation may also be the result of the use of microsatellite DNA markers, which may be polymorphic.


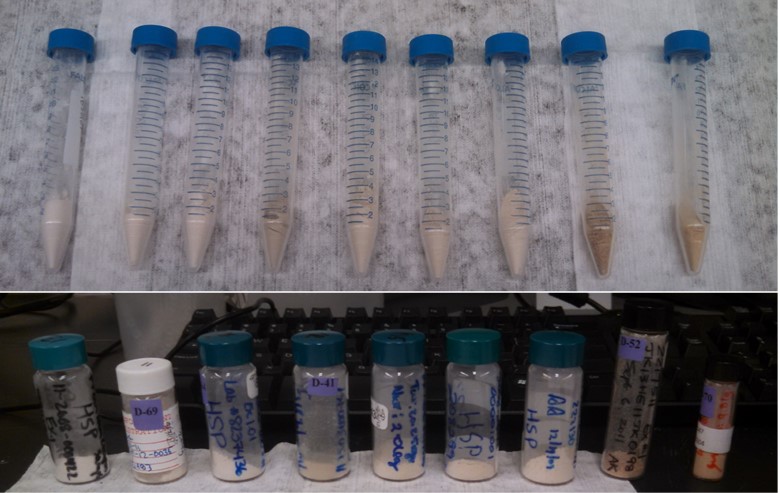


Figure 3A: One gram of various powdered heroin samples.


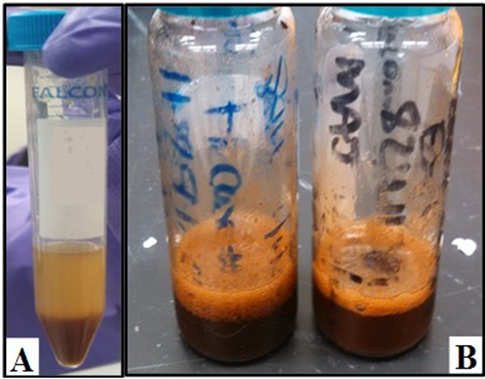


Figure 4A: (A) Sample with an apparent biphasic appearance following the initial incubation at 70°C. Most samples have been in a single phase, particularly the samples with higher purity. (B) Black tar heroin samples were difficult to remove from the vials. Therefore, they were extracted directly in the original vial to maximize the yield. The samples did go into solution but remained fairly cloudy during the entire extraction process up to loading onto the column.


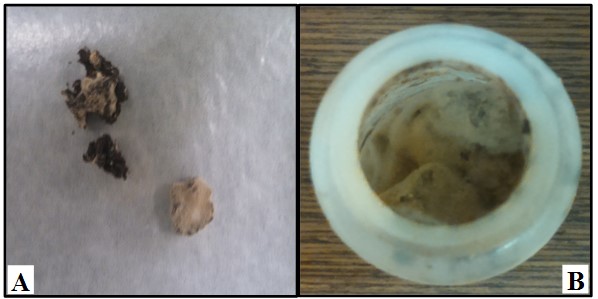


Figure 5A: A moldy opium sample – EE3-2 (A), when extracted had a creamy appearance with a mint-like odor (B-view from above a 15mL vial following the initial incubation). Attempts are made to exclude mold from the DNA extraction process.


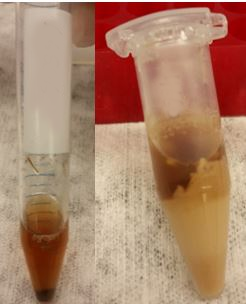


Figure 6A: Pieces of what appears to be plant material stuck on the surface of the interior of the tube and floating on the surface of the liquid (left). An example of insoluble, low purity samples that fail to go into solution despite additional heating (right).
